# A Novel Markov Model-Based Traffic Density Estimation Technique for Intelligent Transportation System

**DOI:** 10.3390/s23020768

**Published:** 2023-01-09

**Authors:** Hira Beenish, Tariq Javid, Muhammad Fahad, Adnan Ahmed Siddiqui, Ghufran Ahmed, Hassan Jamil Syed

**Affiliations:** 1 Faculty of Engineering Sciences & Technology, Hamdard University, Karachi 74600, Pakistan; 2 College of Computing and Information Science, Karachi Institute of Economics and Technology, Karachi 75190, Pakistan; 3 School of Computing, National University of Computer and Engineering Science (FAST-NUCES), Karachi 75030, Pakistan; 4 Faculty of Computing & Informatics, Universiti Malaysia Sabah, Jalan UMS, Kota Kinabalu 88400, Sabah, Malaysia; 5 Cyber Security Research Group, Faculty of Computing & Informatics, Universiti Malaysia Sabah, Jalan UMS, Kota Kinabalu 88400, Sabah, Malaysia

**Keywords:** industrial Internet of things, fourth industrial revolution, intelligent transportation system, traffic density estimation, traffic efficiency, Markov model, connected vehicle, vehicle to everything, dedicated short-range communication, long-term evaluation

## Abstract

An intelligent transportation system (ITS) aims to improve traffic efficiency by integrating innovative sensing, control, and communications technologies. The industrial Internet of things (IIoT) and Industrial Revolution 4.0 recently merged to design the industrial Internet of things–intelligent transportation system (IIoT-ITS). IIoT sensing technologies play a significant role in acquiring raw data. The application continuously performs the complex task of managing traffic flows effectively based on several parameters, including the number of vehicles in the system, their location, and time. Traffic density estimation (TDE) is another important derived parameter desirable to keep track of the dynamic state of traffic volume. The expanding number of vehicles based on wireless connectivity provides new potential to predict traffic density more accurately and in real time as previously used methodologies. We explore the topic of assessing traffic density by using only a few simple metrics, such as the number of surrounding vehicles and disseminating beacons to roadside units and vice versa. This research paper investigates TDE techniques and presents a novel Markov model-based TDE technique for ITS. Finally, an OMNET++-based approach with an implementation of a significant modification of a traffic model combined with mathematical modeling of the Markov model is presented. It is intended for the study of real-world traffic traces, the identification of model parameters, and the development of simulated traffic.

## 1. Introduction

**Figure 1:**
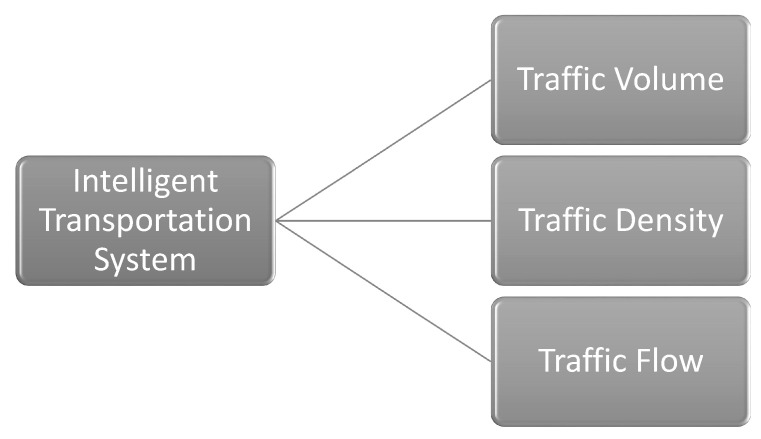
Data-collection aspects of intelligent transportation system.

Transportation plays a vital role in the daily lives of people. According to a prediction [1], by 2030 the amount of traffic in the world is going to increase by 60%. Significant research has been done in the area of intelligent transportation systems in recent years. By deploying advanced data communication technologies, ITS integrates information, communications, and other technologies and applies them in the field of transportation to develop an integrated system of people, roads, and vehicles. It is capable of establishing a huge, fully operational, real-time, accurate, and efficient transportation management system. Traffic flow, traffic density estimates, and traffic volume are the three main data investigation components in ITS. An intelligent transportation system needs real-time information, such as journey time, traffic density, and other factors, in order to make effective control decisions and provide reliable information to users. Figure 1 illustrates the three primary input data that ITS needs: traffic volume, traffic flow, and traffic density. Although the intelligent transportation technologies for various communication systems vary, all of them enable information to be sent based on the vehicle’s data in terms of various data-gathering aspects from the various sources, i.e., traffic flow, traffic volume, and traffic density methods, as shown in Figure 1. When people must spend a lot of time on the road due to traffic congestion, everyone’s lives are impacted. Researchers have been working on categorizing and evaluating traffic circumstances to find solutions to these traffic problems. Traditional traffic-management techniques, such as controlling traffic with wireless sensors, vehicle speed guns, roadside radars, and infrared counters, are ineffective at controlling congestion. The traffic density estimate, in contrast to traditional traffic systems, offers useful information in ITS, such as traffic control, traffic condition, and early prediction, to ease the problem of traffic congestion. Researchers proposed a framework based on these constraints, i.e., vehicles, people, and their external environments connected with associated sensors [2]. There are various simulation tools available for the processing and analysis of these attributes. Modern simulation tools were recommended by research work along with their features. The safety parameters of various commercially available simulation frameworks were discussed by the authors and are also covered in Table 1 of this paper. The authors concluded that a safety microsimulation model is required in heterogeneous traffic circumstances, especially in developing nations after discussing the tools’ advantages and disadvantages [3]. In intelligent transportation systems for traffic management and control, traffic density estimation is used as a form of automated measurement. ITS employs those metrics for the route planning, smart road transportation, proactive traffic regulation, street traffic control, network traffic synchronization, routing, and distribution. For the generation of early warning and autonomous signaling systems, precise measurements of traffic density are required. Intelligent transportation systems use three major classifications: microscopic, mesoscopic, and macroscopic. All these categories are further connected by two continuous and discrete approaches. Mesoscopic models combine both macroscopic and microscopic aspects, i.e., macroscopic and microscopic models. Data for these models are collected via one of three methods, as shown in the Figure 1.

### 1.1. Traffic Flow

Traffic flow is the intersection between roadside units, travelers, and vehicles. This information will impact the intelligent transportation system. Road networks become intelligent when they can predict traffic flow over time, and this resonates with how we live our daily lives. This information attracts researchers from a wide range of domains, including science, statistics, engineering, and machine learning.

### 1.2. Traffic Density

**Figure 2:**
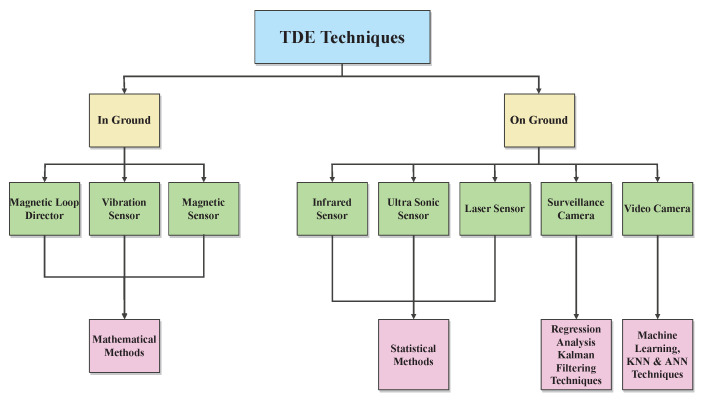
Taxonomy of Traffic density estimation techniques.

Due to its inclusion in microscopic models, traffic density is a measurement that is frequently utilized in ITS. To improve the effectiveness of intelligent transportation systems, the computation of traffic density is a crucial component of predictions used for warnings of systems, roadside networks, and people. The capacity of the driver to choose routes is enhanced by this knowledge. Consequently, a variety of strategies are employed in TDE, as shown in Table 2. By using these methods, we evaluated the aforementioned dimension as shown in Figure 2. The procedures used in those methods were also described in Table 3, along with connected urban and highway scenarios, data-collection strategies, and the algorithms they had implemented for vehicles, which are more concentrated in urban areas with a higher population density than in highway areas with a dense population.

**Figure 3:**
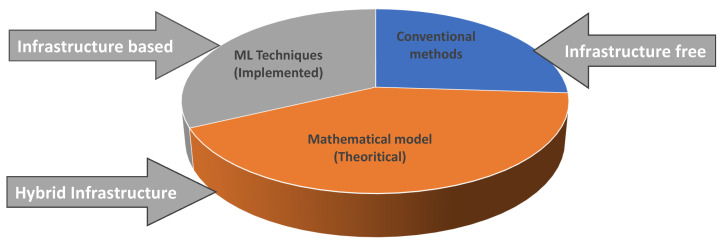
Distribution of TDE Techniques.

Figure 3 illustrates how these methods for estimating traffic density are based on three different proportions: conventional methods, mathematical models, and machine learning models. It shows how various TDE methods are distributed. The TDE approaches, which were based on the method used to estimate traffic density, are further explained in the Figure 3. However, these methods were created to estimate traffic density for a number of purposes and applications. Applications that employ density estimation techniques fall into three categories: conventional, mathematical, and machine learning methods.

### 1.3. Traffic Volume

Traffic volume is a way by which the volume of traffic or number of cars traveling on a road section at a specific period of time can be found. There are numerous volumes studied in traffic engineering, including daily volume, hourly volume, and peak hour volume. Additionally, the volume of a day or hour might change significantly based on the day of the week or the hour of the day.

### 1.4. Research Questions

The following is how the research questions for the studies are put together.

1. What are the dimensions used for identifying traffic density estimations?
2. What evaluation tools are available for analyzing suggested methods?
3. How do TDE algorithms analyze various traffic behaviors to process the road network?
4. How to choose a suitable approach to investigate an efficient traffic analysis technique?

There are several ways by which to estimate traffic; however the techniques employed nowadays are more useful and frequently involve ML-recognized algorithms, which are typically used for real-time estimations. The Background section of Table 1, Table 2 and Table 3 includes a full explanation of the answers to the aforementioned questions.

We present the concept of a Markov approach for estimating traffic density in the road model and mathematical explanation, which illustrates the behavior of a single vehicle and depicts the traffic on the entire road network. We use the microscopic traffic simulator SUMO to build a traffic network with stochastic traffic demand and real values for traffic density in order to assess the effectiveness of the Markov traffic model. The SUMO-generated traffic demand is provided to the Markov traffic model, and the link state will change to the mode with the highest transition probability on each iteration. Because traffic conditions might fluctuate from day to day, it is highly convenient to estimate future traffic conditions based on initial traffic conditions. Due to the simplicity of this model, once it has been expanded, the public can use it to investigate traffic estimation on their local road network. The counts of each type of vehicle accessing a road segment are also shown as nodes 0, 1, 2, 3, and 4. The study’s main goal was to create an effective traffic analysis model that could be used to simulate traffic in a system of roads. To depict the traffic system, mathematical traffic flow models are also typically utilized. They are mathematical descriptions of the complicated traffic systems used to characterize and predict traffic behavior in the future. This research aims to develop a Markov-based mathematical method for estimating density in mixed traffic conditions.

### 1.5. Motivation and Contribution

Vehicles increased traffic in cities enormously. These produce traffic bottlenecks, which cause air pollution, personal health issues, lost productive labor time, and economic losses. Because of this, and considering the inconvenience that traffic causes, managing traffic with intelligent systems and technology is necessary. As traffic density on a route is an excellent indicator, quick and reliable real-time estimation of traffic density is critical for traffic control. This research is being carried out by employing intelligent technologies to estimate traffic density. By utilizing the current traffic state as well as mathematical modeling based on the Markov process to predict the future traffic state while taking into consideration a predetermined road structure with a minimum of 0–4 vehicles and a length of 1 km that is connected to a distinctive roadside unit. In contrast to statistical analysis, these systems use artificial intelligence to determine traffic density. These are equipped with traffic data and used to estimate the traffic in real time. In this research, different types of conventional methods are compared, and a novel framework is proposed to examine the usefulness, appropriateness, and robustness of the proposed framework, which is analyzed through simulations.

The structure of this essay is as follows: The backdrop of the research findings is presented in Section 2. In Section 3, a model for estimating traffic densities by using mathematical modeling was proposed. In Section 4, model simulations are carried out. We established a vehicle tracking in Section 5, and the results of such tracking are shown in Section 6. Section 7 presents the findings and related discussion. Future research directions are presented in Section 8 as the paper’s conclusion.

## 2. Related Work

Numerous academics have made good progress in the past in increasing traffic control and analysis observations for traffic density analysis. For estimating traffic density, magnet loop detectors, and monitoring cameras are used. These traditional methods include wireless sensors, speed cannons for vehicles, roadside radars, and infrared counters. Magnetic loop detectors require a lot of hardwiring technology-based physical work. In addition, its setup and maintenance are highly expensive. Therefore, these technologies are inappropriate for use in large-scale and real-time applications. There are several approaches that can be employed to use the data gathered by cameras or sensors. Vision-based traffic monitoring mechanisms choose the images and videos to collate data. Studies of data extraction focused on traffic state estimation and modelling make use of stationary sources. To increase the functionality of traffic density estimation systems, many studies are discussed. For estimating traffic densities, three basic categories are typically utilized. By detecting each vehicle, such as a bus, bicycle, car, and truck, the authors of [18] proposed these approaches based on loop detectors and sensors for building the sensor’s structure. In [19], the authors presented a detection system based on a radar probe with the integration of proposed algorithms. Studies in [20,21,22] described a method for traffic density estimation that focuses on traffic surveillance cameras and gathered real-world information in the form of photographs. This approach improves traffic density prediction methods because they can track vehicle classification and identification with greater specificity. For calculating the traffic density by a fixed traffic detector, further methods are also used [23]. Because there are primarily two major kinds of traffic density, as shown in Figure 2, several on-the-ground procedures are also employed. The literature review revealed that different sensors, approaches, procedures, and input data are used by researchers for various ITS applications. Sensor technologies play a significant role in the data-collection process that takes place during vehicle-to-vehicle (V2V) and vehicle-to-infrastructure (V2I) communication. The sensors provide traffic-related data such as speed, volume, density, individual vehicle classification, and much more. Typically, sensors are placed adjacent to or above the route of interest, and in certain instances, a single sensor can be utilized for many lanes. Installation and upkeep of these sensors are simplified. Off-road sensors are mobile sensors that can be utilized on GPS-equipped vehicles. Installations of cameras and various sensors along the roadway can detect the presence of automobiles, and a global positioning system (GPS) can monitor the vehicle throughout its trip. Moreover, vehicle detection is based on image and video processing by employing both static and dynamic cameras. Traditional traffic monitoring techniques include invasive sensors such as inductive loops, pneumatic tubes, and piezoelectric sensors. However, these devices are expensive and difficult to maintain, as road surfaces should be removed in order to place them, and installation disrupts traffic. Based on the viability of implementations and regions, sensors are selected. Each sensor, as indicated in Figure 2, operates in a distinct manner and corresponds to a different detecting method, as shown in Figure 2.

These magnetic loop detectors allow the detection of vehicles in heterogeneous, less-lane traffic. In [19], the authors proposed a loop sensor detector system for all-size vehicles. Their proposed solution utilizes available track capacity to meet the requirements for sensing heterogeneous and near-linear traffic circulation. Road vibration sensors are used to accurately calculate parameters like speed, direction, and vehicle type with particular relevance due to their energy potential through piezoelectric films. In [26], the authors work on piezoelectric acceleration sensors to evaluate the amplitudes and frequency ranges, in which vibrations occur according to established measurement principles in microelectromechanical system (MEMS) accelerometers. A substantial amount of information about traffic situations is collected from magnetic sensors. Many studies have explored the use of magnetic sensors and other types of traffic information for vehicle counting [27]. Magnetic sensors’ characteristics include low cost, energy efficiency, small size, wireless connectivity, and meteorological independence. The traffic density estimations are also obtainable, in addition to the on-ground techniques. On-ground methods are used mainly, as they are the future of urban traffic estimation. Vehicle data is often compiled by using high-pole cameras. Sensors are essential for measuring the route’s traffic density. For gathering traffic information, various types of sensors are used by the authors in [28]. Multiple sensors capture the energy generated by vehicles and the road surface and transform it into electrical impulses by using infrared radiation. For traffic prediction and differentiation of traffic congestion levels, the authors employed the 14 on-road sensors named (D1–D14) at multiple spots. In [29], the authors discussed the use of surveillance cameras for traffic data collection, which uses more advanced sensors, such as surveillance cameras. For deriving the traffic information from video sources, these devices use computer vision techniques. The number of vehicles is simultaneously detected and counted. Traffic density is measured by tracking and displaying the number of vehicular nodes in the road camera image at almost the same time. Many cities have low-cost closed-circuit television (CCTV) surveillance systems (CCTV). To date, only a few studies have attempted to automate the processing of surveillance video data for traffic analysis. They are rapidly expanding and usually include a range of cameras with varying resolutions and mounting points. In [35], the authors proposed a method for estimating traffic flow for counting and categorizing vehicles based on their moving directions. They divide the problem into three subtasks to achieve this goal: vehicle identification, vehicle tracking, and vehicle path estimation. In the related work mentioned above, the authors discussed and implemented traffic density with different perspectives. The authors employed a conventional method for estimating the traffic density by using underground loop detectors [18,26]. The new techniques for estimation of traffic density were found in the literature by using the deep learning method [5]. These techniques are used to estimate traffic density and weather conditions to forecast traffic conditions. Surveillance cameras were used successfully in 2009 to estimate traffic density in Istanbul, Turkey [22]. Several other methods for estimation include the Highway Capital Manual (HCM), Kalman filtering, variance blocks, and kernel density [2,19,26,32]. However, those are not implemented due to the basis of statistical theories.

- There are shortcomings in the sensor-based estimation methods because of the sensor’s range limits. By increasing the range of these sensors, some of these shortcomings are removed on a small scale [2].
- Another method used for traffic estimation is based on vehicle trajectory detection [37]. However, this method has limitations due to vehicle background obstacles, such as traffic signals, buildings, shadows, etc. These limitations are reduced by implementing the light detection and ranging (LIDAR), radar, ultrasonic, and infrared (IR) sensors [26].
- Image- and video-based detection methods are also used for detecting vehicles to achieve traffic density estimation. However, there are also limitations in the form of blur images and noises in the background. Many authors proposed using methods like receiver operating characteristic (ROC) analysis to overcome these limitations [42]. Researchers have been working on traffic density features and have differentiated three main categories: connected autonomous vehicles (CAV), human vehicles (HV), and a combination of both, i.e., hybrid vehicles. These categories are available for the enhancement of traffic density indicators in ITS.

The Kalman filtering (KF) and kernel density estimation methodology is likely the most extensively used technique for traffic density estimation. The authors of [2,3,18,20,26,27,28,32] have worked on this technique by using the typical data-collection techniques of loop detectors, LIDAR, and vehicle sensors. Magnetic loop detectors and other hardwire-specific technologies need a considerable amount of physical effort. Additionally, it is exceedingly expensive to install and maintain. This is why this type of technology cannot be utilized on a wide scale or in real time. Computer vision and AI approaches are the future of traffic monitoring in urban areas. The authors of [20,21,23,25] estimated traffic by using computer vision algorithms. In [27,28,29,30,31,32,33,34,35], the authors counted automobiles by using traffic cameras and live video feeds.

The estimation of traffic parameters for a number of different methodological approaches is applied for the calculation of traffic density. In addition, research was conducted into the urban and highway-based estimating systems, as well as the relevant design strategies for TDE. The authors of [25,26,27,28,29,30,31] worked on urban scenarios, whereas the authors of [24,25,32,34,39,41] presented estimations based on the highway scenario. In reference, Live data was utilized by the authors in the sources in order to carry out the distributed state observers of TDE.

Traffic models are microsimulation models that are based on the representation of the behavior of each driver regarding many features’ car following, gap acceptance, and lane choice rules.

Table 1, Table 2 and Table 3 show the different available approaches focusing on traffic density estimation methods and achieving related sustainability characteristics for transportation sys-tems. These include methodologies that address V2V, V2I, and vehicle-to-everything (V2X) communication in urban and highway areas.

Traffic density is somewhat correlated with neural network techniques, and artificial neural networks (ANNs) are a subset of artificial intelligence that may address complex problems in dynamic environments by providing clear and concise recommendations. In recent years, artificial intelligence (AI) has gained prominence as a viable solution to resource-constrained sensor networks’ complexity, scalability, and decision accuracy constraints. AI has been applied in a variety of applications, including MIMO networks, time series prediction, image processing, and WSNs. Authors of [43] proposed an AI-based (MIMO) strategy with adaptive neural network transportation systems, as well as energy saving in ITS systems for vehicle and infrastructure management. Relative work is also done by the authors of paper [44], who presented a neural network-based AI-based energy-efficient routing protocol for an intelligent transportation system, and they tried to overcome the problems of an intelligent transportation system by adding quick communication between clusters and by combining DAI with self-organizing maps (SOM). This study provides new methods for improving energy efficiency. It also reduces the overall network’s energy consumption and computational burden. To increase the network’s lifespan, their proposed strategy prioritizes nodes with remaining energy above a predetermined threshold value. Considering that security is the primary concern in vehicles’ communication, some effort has been done to improve vehicle security, such as proposing a lightweight, blockchain-based safe architecture for IoVs that offers strong authentication and communication security [45]. To address the problems of cross-trusted authority authentication in the Internet of vehicles (IoV), the authors of [46] proposed an authentication framework using blockchain technology. Their analysis is employed to reduce the execution times and improves in understanding the impact of feature selection [47]. In intelligent transportation systems, the conventional cooperative ITS(C-ITS), is another term, and as compared to V2X it employs the same messaging protocols, but they use a separate radio communications technology, and the real-world application is the same for both standards for V2X and C-ITS. V2x employs the DSRC, while C-ITS employs a separate communication system. While merging the two, C-V2X is still in the final stages of testing. V2X is sensor-based, whereas DSRC and C-V2X are rooted in separate technology, resulting in fundamentally different operational techniques. DSRC is a wifi-derived protocol that is optimized for affordability and simplicity, and it allows dispersed operation by default. C-V2X, which evolved from LTE. In this research, we have used DSRC-based communication.

## 3. Proposed Model

**Figure 4:**
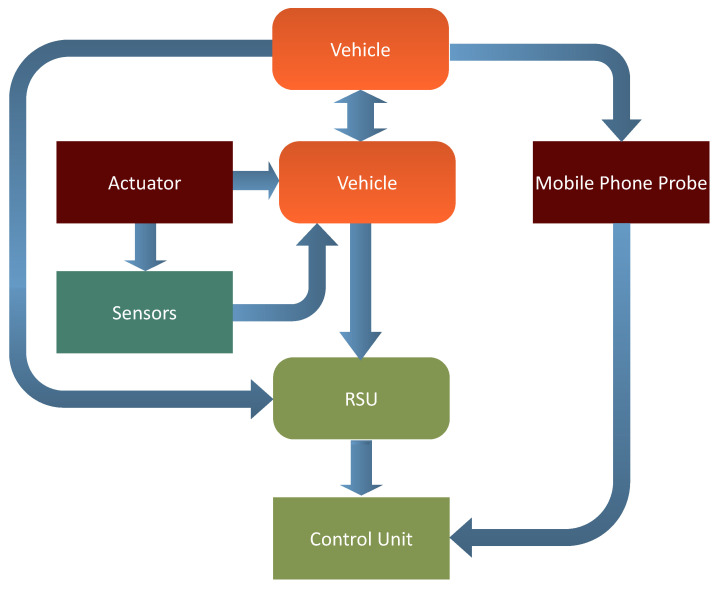
Schematic block diagram of proposed model.

The development of the IIoT and the use of ITS necessitate a series of procedures for architectural communication. This model depicts the V2X architecture in its entirety. Each vehicle has an onboard unit (OBU) equipped with wireless communication capabilities. The OBU, in particular, provides the interfaces required to estimate vehicle density by using data obtained via node to infrastructure communications. Infrastructure equipment, such as RSUs, is necessary to implement the V2I component of our system. Vehicles will benefit from RSUs in particular by receiving updated services. Additionally, the location of RSUs enables the cars to communicate with one another. A block diagram of the proposed model is shown in Figure 4. In this schematic block diagram, we have two communication modes i.e., V2I and V2V. That is the reason we have two blocks of vehicle in the diagram. Figure 4 depicts the vehicles’ behavior by using a flowchart. The data collected from the actuators and sensors is sent to the vehicle, and the vehicle transfers this information to the RSU for further processing and to other cars within the range. Through V2I and V2V communication, vehicles will transmit and receive messages or beacons with the same event ID from the roadside unit and neighboring vehicles.

**Figure 5:**
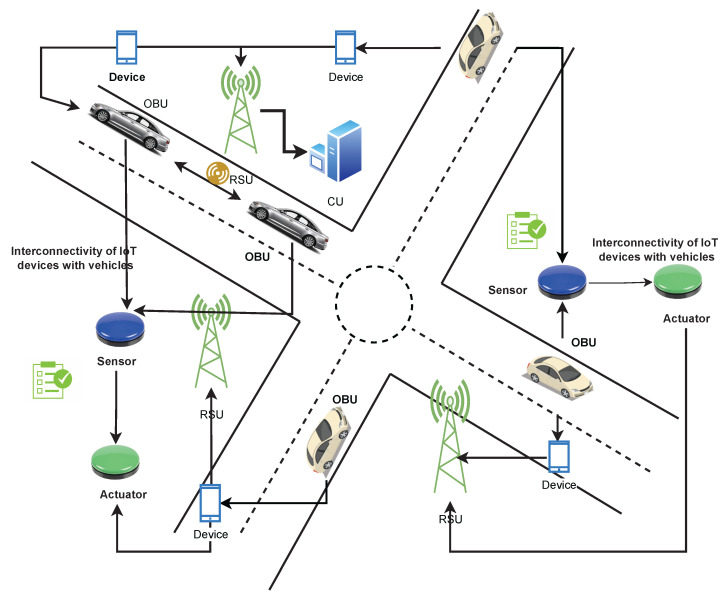
Proposed model with V2I communication.

Figure 5 shows the proposed ITS system’s components: transportation management centers (i.e., control units (CU), field equipment (e.g., radio towers, wifi connectivity, and mobile equipment connected to vehicle movement), vehicles equipped with ITS equipment (e.g., buses, commuter trains, and cars all represent logistics in industrial 4.0), and traveler equipment (e.g., mobile devices) that meets the stakeholder needs. The phones transmit their location data to the mobile phone network regularly. As the car moves, the mobile phone signal acts as a probe. The probe data is provided by the onboard mobile unit, which is connected to the sensors and actuators. Roadside units (RSU) collect data from each vehicle, including traffic congestion and the vehicle’s location. This information will be sent by the RSU to the control unit, which calculates the estimation. A RSU is a transceiver for dedicated short-range communications (DSRC) positioned across a road. This model enables V2V and V2I communication, as well as the collection and exchange of data between vehicles and roadside infrastructure. Vehicles, on the other hand, can be aware of the number of vehicles in their vicinity, because although RSUs can supplement their information on traffic distribution with density information given by vehicles.

### Markov-Based Mathematical Modeling

Markov is a mathematical model for understanding the process of creating evaluation state sequences at random. Currently, Markov acts as a very robust and heuristic for matching state sequences that are consistent with the randomness, uncertainty, and complexity of actual transportation systems. This is owing to the increasing expansion of big data and machine learning. The Markov is an illustration of a discrete-time stochastic process. In such processes, the probability of every future event depends only on the current state and is independent of the states that came before. Markov chains are used in a wide range of scenarios because they can be built to model various real-world processes. For their useful properties such as states, i.e., state transitions. It consists of a set of states and the conditional probabilities of their transitions from one state to another state. Markov provides a long-run probability for traffic estimation. In a Markov model, the system’s future behavior is based only on the present. Therefore, it is highly appropriate for us to predict the traffic condition with Markov. Many authors worked on Markov for the future prediction as [1,47,48,49,50]. This is due to the fact that the Markov model predicts that the conditional probability does not change over the passage of time. This model is the only stationary vehicle distribution on the road network. The model’s significance is that it can estimate the probability of a state being in a given traffic congestion state depends only on the current state. The model provided successfully calculates the probabilities of a specific road section being in a given state of traffic congestion for traffic density. As benchmarks, some stochastic process models have been proposed. The authors of paper [48] suggested an analytical method for estimating traffic delays at unsignalized intersections with Markov process. Coupled HMMs were deployed by the authors of [1] to simulate freeway traffic and estimate the traffic speed.

A hidden Markov model based method also used to address the density estimation on a multilane road segment in [49,50]. In another study, the authors overcome the limitations of adaptive K-means clustering and use the pattern by using regression models. In this study, the Markov model was employed to identify traffic patterns to predict the congestion [51,52]. We proposed the Markov approach because it does not require a lot of data, and it can predict the future state by using only the data from the current state. Whenever one event’s occurrence has no effect on the probability of the other event occurring, two events are said to be independent. This model’s aim is to predict when and where there will be heavy traffic in a traffic network. The model’s reliance is on specific traffic segments rather than specific vehicles or traffic flow. Suppose that the probability of the transition for the state *i* to the state *j* is the transition probability from the state *i* at the time *n* to state *j* at the time n+1 for every pair of states *i* and *j* is represented as. Xi is the observed variable for present and future states and describes the system’s dynamic behavior.

(1) Pn,i,j=Pr(X(n+1)=j|X(n)=1)

Here, the Markov states with respect to time can be stated as follows:

(2) $$X\left(\;t_{1}\right)\;\to X\left(\;t_{2}\right)\;\to X\left(\;t_{3}\right)\;\to X\left(\;t_{4}\right)\;$$

Equation (2) represents the current and future states. The movement of time $t_{1}$ to time $t_{2}$, is only dependent on the last previous state, i.e., only the current connected state. Therefore, the state equation for traffic density for projected vs. actual traffic conditions also provides the real time information, as shown in Equation (3).

(3) TD(i,j)=X(t)+X(t+1)X(t+2)

We designed a scenario that calculates the traffic density with its location and mobility as well as traffic flow. Due to the mobility path loss model that is also used in it, in Equation (4), a communication link is defined for one vehicle. We have

(4) $$TD\left(\;pl\right)\;=\left\{\begin{array}{c} 1,pl\leq p0\\ 0,pl\geq p0 \end{array}\right.$$

where TD(pl) represents the probability of connection between the cars, and pl is the path loss from the measured data. Path loss can be calculated based on the distance between the vehicle and the roadside unit, p0 is a benchmark for path loss. If pl is less than p0, connections are established successfully; otherwise, connections are unsuccessful. The relationship between $TD_{avg}$ and traffic flow $TD_{f}$ is shown in Equation (5) for 1 km of road length. We have

(5) $$TD_{avg}\left(1\;\mathrm{km}\right)=TD_{f}\left(\;1-\frac{TD_{min}}{TD_{max}}\right)\;$$

where $TD_{avg}$ is the average speed of traffic, $TD_{f}$ is the flow of traffic, and $TD_{max}$ is threshold density, which is assuming 25 vehicles per km. To develop the Markov-based traffic density equation, we assumed that the flow (volume) of traffic on the road is 25 vehicles per unit time. This assumption enabled the development of the model to calculate traffic density in terms of the Markov approach. The minimal value, or $TD_{min}$, is the least possible value. The density of estimation $TD_{avg}$ is subject to Bernoulli distribution as shown in Equation (6):

(6) $$TD_{avg}=1-P\left(\;TD_{f}\left(\;1-\frac{TD_{min}}{TD_{max}}\right)\;\right).\;$$

The mutual exclusion of a few vehicle types is also done in this modeling; we have excluded bikes from the traffic density calculation from the range of 1 km as shown in the Equation (7):

(7) $$TD_{avg}^{b}=P\left(\;bik_{b}|TD_{avg}^{b}\right)\;=\frac{\left(\;1-TD_{min}\right)\;-bik_{b}}{1-TD_{max}}$$

$TD_{avg}^{b}$ is the mutually exclusive event of the bike, and is the probability of the bike over ${TD_{avg}^{b}}^{\prime }$.

The probability of TD with discrete time Markov chain with interval indexes is t=0,1,2,3,n. Consequently, the Markov property is shown in Equation (8):

(8) $$P\left(\;TD\left(\;t_{n}\right)\;\right)\;=x_{n}|X\left(\;t_{n-1}\right)\;=X_{n-1}$$

The multistep transition probability of the Markov chain model is shown in Equation (9):

(9) $$\int P\left(\;X_{n+2}|X_{n+1}\right)\;)P\left(\;X_{n+1}|X_{n}\right)\;)dX_{n+1}$$

According to the Markov chain property for the finite state is shown in Equation (10):

(10) π=πp

Finally, calculating the traffic density using a Markov chain model can be calculated by using Equation (11).

(11) $$TD_{avg}=TD_{f}\times \pi$$

Equations (1)–(3) and (8)–(10) are the general description of Markov property and Equations (4)–(7) are derived for traffic density.

## 4. Model Simulation

OMNET (Available at https://omnetpp.org, accessed on 30 October 2022) is used in a V2I environment in conjunction with the simulation of urban mobility (SUMO), available at https://www.eclipse.org/sumo, accessed on 30 October 2022) to establish communication. The map is selected initially, and is followed by the number of vehicles as well as the route. The network is imported from the OpenStreetMap (OSM) database. This map covers an approximate area. This dataset is exported to SUMO and then imported into Omnet++ to the Cologne map. The display and storage of map data is supported by a simple and powerful idea. We used the road traffic simulator SUMO to generate vehicular traces, and then we examine the connectedness of the vehicular network. The network is then populated with random traffic. We assume the conception of a “node”, by considering any vehicle as the general vehicle in which communication passes from RSU to node, node to RSU, and from one node to another node. However, associating vehicle detection with a sequence of consecutive frames is achievable. Vehicles start moving immediately and can stop by any traffic light triggered at a particular position from where the RSU transmits a message to vehicles regarding the traffic jam.

**Figure 6:**
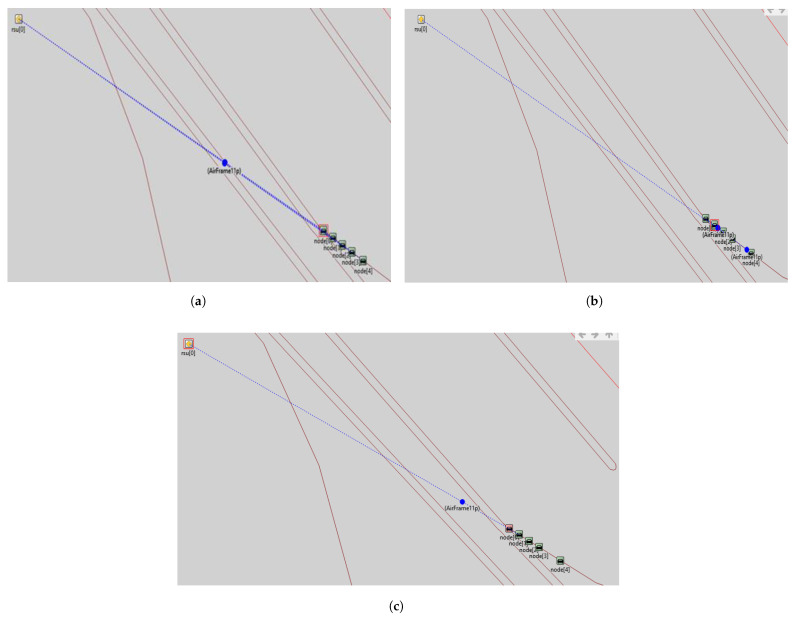
Communication modes. (**a**) Communication from RSU to node. (**b**) Communication from node to node. (**c**) Communication from node to RSU.

According to the proposed model in Section 3, various IoT devices are interconnected with each other in a simulation environment, as shown in Figure 6a–c, vehicles communicate messages to other vehicles shown in Figure 6b, as well as to RSU Figure 6c. The RSU can send a message to all vehicles in his zone as shown in Figure 6a. Based on RSU analysis, Signals are transferred to the control unit for data transmission. The control unit receives information from different RSUs and sends it to other RSUs in its region, e.g., RSU to node, node to node, node to RSU, and RSU to CU as mentioned in Figure 6a–c. These Figure 6a–c are based on the vehicle’s ability to interact with the infrastructure and conversely. Communication takes place between vehicles dependent on whether they are within or beyond the coverage area of which roadside equipment. RSUs based on LTE are considered fixed units, whereas cars are considered dynamic modules. In these figures, each vehicle can communicate with RSU throughout its given traveling time.

**Figure 7:**
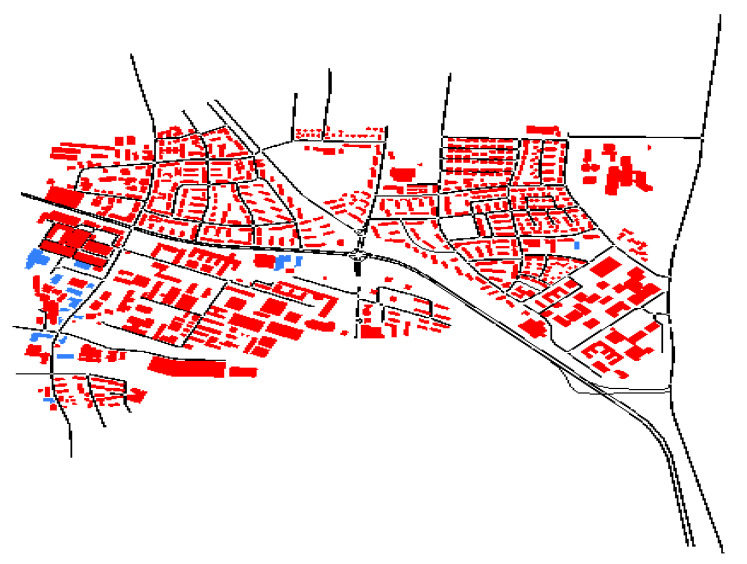
Imported map interface with SUMO for vehicles’ routes.

Figure 7 shows the imported map interface with SUMO for vehicles’ routes. A map can be generated to create an environment simulating road traffic in a real-world route by using the OpenStreetMap (OSM) (available at https://www.openstreetmap.org, accessed on 30 October 2022) is a collaborative initiative that allows anyone to contribute to the creation of a free map of the world. The data is made available under the terms of the Open Data Commons Open Database License (ODbL) (available at https://opendatacommons.org/licenses/odbl, accessed on 30 October 2022). A simple yet powerful concept underpins the display and storage of map data. We started with the OSM map from Porto (Porto is the second largest city in Portugal), and created a graph which used the same region of interest as the dataset filtered. The nodes in the graph are specific nodes from the OSM file. Only certain sorts of way nodes could be collected after we ran a filter on the OSM dataset, because only needed nodes that could be reached by vehicle.

**Figure 8:**
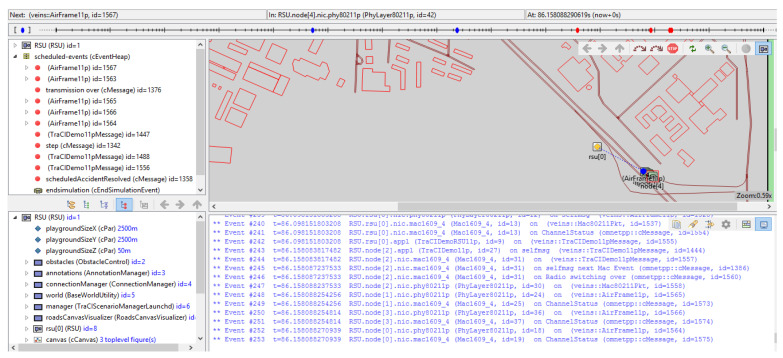
Simulation scenario on OMNET++.

Figure 8 shows the simulation scenario environment in OMNET++ with results. OM-NET++ is used in order to simulate mentioned scenario in realistic wireless communication scheme. SUMO and OMNET++ is integrated into the framework veins. Initially, traffic-related vehicle information (such as start time and location, stop time and position, origin, destination, maps, etc.) is generated in SUMO, then imported to OMNET++. In the network simulator, all vehicles are considered nodes. Veins modify the scenario of the automobile in OMNE++ if any modifications are made to SUMO and vice versa.

Table 4 shows the events during the specified time duration with Beacon ID and packet ID used in OMNET as packet recognition. There are five nodes (vehicles) used in this simulation. Various nodes of communication are employed. For the proof of concept, we create our setup with five nodes, the limitation of our technique is that we have only employed a small number of nodes; in future work, we can use more nodes. On each node, an application is running to generate packets at regular intervals. The reason for the minimal number of nodes is that more nodes will send more acknowledgments to the infrastructure and to the other nodes, whereas a higher number of nodes will take more time and there will be more waiting to conduct and transfer the data to the control unit and send it in order to avoid collisions for the proposed implementation. We have considered five nodes from a 250-s time for two roadside units. If we consider more vehicles, we would have to add more road length with the more RSUs for a specified communication range and other components as well. There is no fixed point for nodes. We can increase the number of nodes by increasing all parameters. The parameters associated with the characteristics are the primary concerns of the performance analysis. In this scenario experiment, these parameters are used, and they are displayed in Table 5. Each experiment set’s simulation time is 250 s for the five nodes. Many vehicles and road attributes, including the list of lanes in the scenario, the form of the lane, the edge ID holding a specific lane ID, the length of the lane, the maximum speed allowed by the lane, and the mean speed allowed by the lane, can be accessed or updated by using TraCI.

## 5. Vehicle Tracking

Tracking of vehicles is obtained by considering the speed, position, and direction in a highway scenario. SUMO is used to implement and track vehicles. We have considered some parameters for tracking, i.e., vehicle position, and speed fluctuations of each vehicle; it also simulates the acceleration of each vehicle. At the same time, the number of beacons is sent to the RSU from each node when traveled within the RSU range with a fixed time interval. The parameters associated with the characteristics are the primary concerns of the performance analysis. In this scenario experiment, these parameters are used, and they are displayed in Table 5. Each experiment set’s simulation time is 250 s for the five nodes. Many vehicle and road attributes, including the list of lanes in the scenario, the form of the lane, the edge ID holding a specific lane ID, the length of the lane, the maximum speed allowed by the lane, and the mean speed allowed by the lane, can be accessed or updated by using TraCI. TraCIScenarioManager and TraCIMobility modules of veins has the ability to run traffic simulation. To implement vehicle tracking additional commands in TraCICommandInterface.cc, TraCIMobili-ty.h files are used. Following are the commands used in simulation.

Begin

TraCICommandInterface()

$VAR_{T}RACK_{V}EHICLEvariableId;$

$TraCIBufferbuf=conn.Query(CMD_{S}ET_{G}UI_{V}ARIABLE,TraCIBuffer\left(\right),variableId,$

nodeId)

ASSERT(buf.eof())

End

Begin

commandTrackVehicle()

getcommandInterface.setVehicleTracking.getExternalId())

End

**Figure 9:**
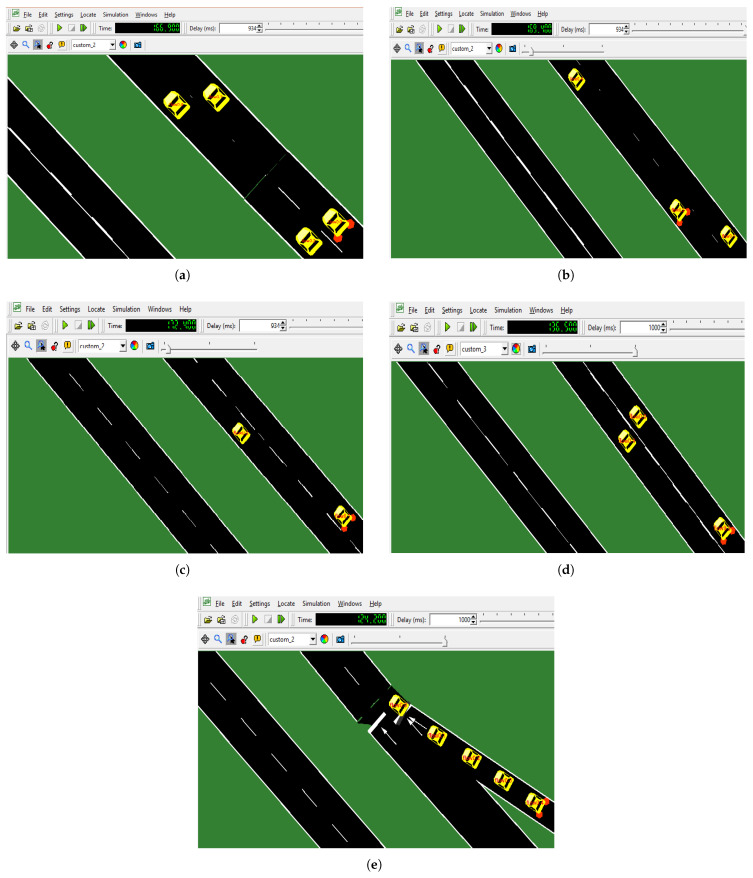
Tracked vehicles in SUMO. (**a**) Tracked vehicle 0 in SUMO as node 0.0. (**b**) Tracked vehicle 1 in SUMO as node 0.1. (**c**) Tracked vehicle 2 in SUMO as node 0.2. (**d**) Tracked vehicle 3 in SUMO as node 0.3. (**e**) Tracked vehicle 4 in SUMO as node 0.4.

We investigate the tracking of five vehicles on a real road network, as shown in Figure 9a–e as (node/flow 0.1–0.4). Specific items can be chosen to track associated parameters, such as a vehicle’s current route, position, or speed. Each vehicle is tracked in its own time by articulating it in circles independently. The individual circle on the vehicle is constantly tracking along the journey.

## 6. Tracking Results

In this evaluation, number of tracked vehicles calculated from the number of messages transferred between vehicles to the road side units. All the five vehicles are routed through the same route. In order to achieve the highest tracking accuracy, we selected the maximum continuous tracking time as a measure. The tracker first builds a traffic lane for beacons that shows throughout the scan. Then, by following intervals, it allocates beacons to the established routes and may initiate new tracks. The high peak tracking duration percentages are utilized as a tracking accuracy metric. The tracking system automatically builds a set of tracks for beacons that appear throughout the scan. Then, in following time steps, it distributes beacons to the established tracks and initiate new tracks. Vehicles are classed into a variety of groups based on their speed vectors. As a result of the tracking, it simulates the acceleration, vehicle position, and speed fluctuations of each vehicle. Because we consider monitoring and tracking on each vehicle, the tracked vehicle maintains a steady speed on the highway. The vehicle’s speed is the primary parameter that governs its dynamics.

**Figure 10:**
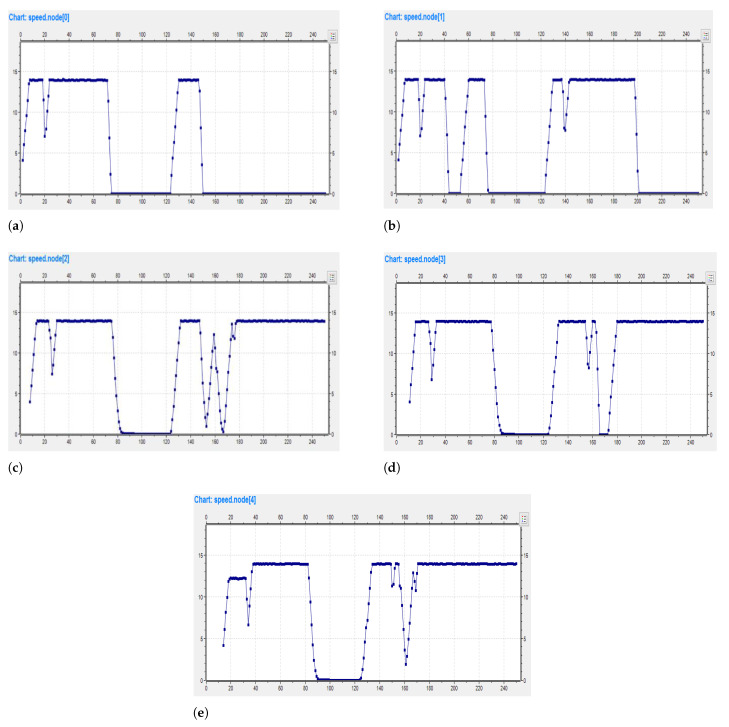
Speed graphs of Vehicles. (**a**) Vehicle 0 Speed; (**b**) Vehicle 1 Speed; (**c**) Vehicle 2 Speed; (**d**) Vehicle 3 Speed; (**e**) Vehicle 4 Speed.

The graph in Figure 10a–e depicts the relation between individual vehicle speed and time. The speed of the tracked vehicle reduces substantially as tracking increases. On either side, the value of speed is low, so there is a high likelihood of successful tracking as shown in the figures.

Table 6 illustrates the differences in the number of vehicles generated for the highway scenario. The three methods further achieve a stable output for the mean vehicle acceleration with a standard deviation. VACaMobil [53] is used to create the network with the necessary number of vehicles. It also includes the specific min and max number of vehicles by using the standard deviation feature, as these vehicles are the primary simulation objects and have numerous attributes, such as the current speed, position, and acceleration. Table 6 values represent the average number of vehicles, their standard deviation, and their continuation over the simulation time.

## 7. Results and Discussion

**Figure 11:**
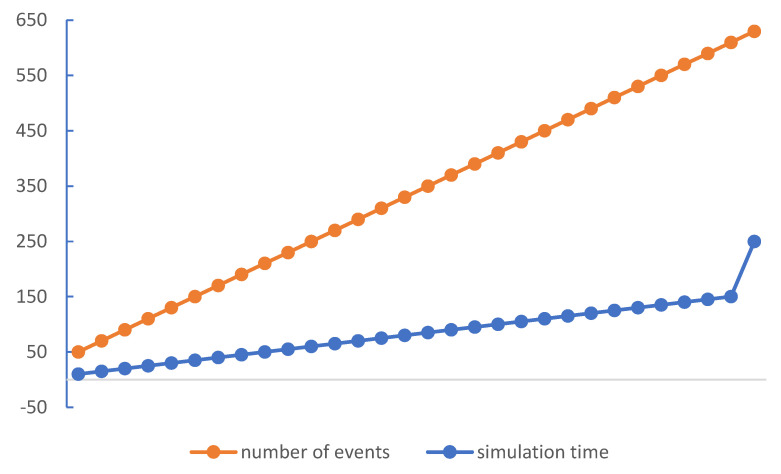
Number of beacons sent vs. simulation time.

**Figure 12:**
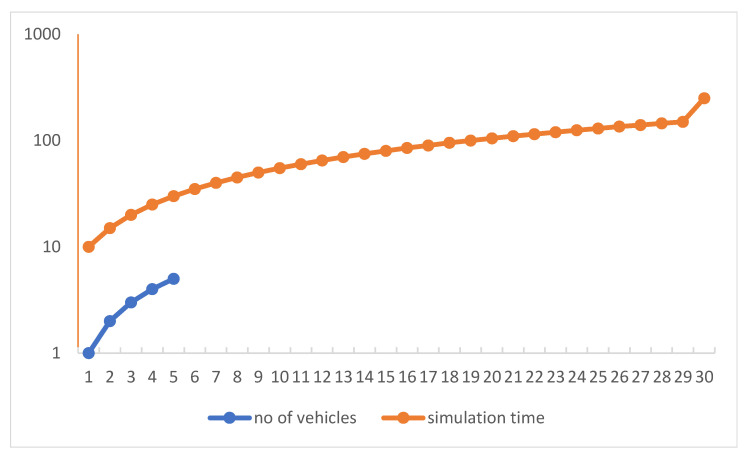
Number of vehicles vs. simulation time.

In this work, we investigate the modeling of traffic density by using the Markov model approach. Moreover, we successfully implemented our model in the SUMO simulation environment by using Omnet++ with the vein’s framework (available at https://veins.car2x.org/download/, accessed on 30 October 2022) that is linked and evaluated for the model’s functionality. Moreover, it enables the implementation of dynamic broadcast propagation techniques, thereby enhancing the communication capacities of vehicle networks. The proposed architecture makes it possible to use effective ways to reduce traffic congestion because it makes easy for cars to act quickly. Moreover, this makes it possible to use model-based broadcast propagation techniques, which improves the ability of vehicle networks to communicate with cellular. The number of cars in this implementation moving in a range of one roadside units’ network that is represented in Figure 8. Figure 6a–c shows the numbers of nodes (cars) enters the simulation environment one by one. The simulation is set up with a fixed number of five cars, and it finishes when the simulation time reaches the given 250 s, as seen in the Figure 11 and Figure 12, which show the number of beacons sent vs. simulation time and number of vehicles vs. simulation time, respectively. These figures depict the amount of time spent in execution that was required to complete the 200-s simulation. We allowed the simulation of the allocated amount of time to run, during which we monitored the number of packets or events that were generated in the designated area, as well as the relationship between the rate of time spent running the simulation and the total number of nodes. Once the beacon has entered the region, all of the nodes in the immediate vicinity will restart sending beacons at a rate of once every second for the duration of the simulation. An arrangement of this aims to establish whether or not the simulation framework is able to continue operating despite the presence of the produced traffic. In this simulation scenario, vehicles travel on premeditated roads determined by SUMO (the model treats all nodes identically) and vehicles that move at unpredictable speeds. The node moves from one spot to another with variable speed and in the same direction as all other nodes. Each entity has a mobility submodule that is in charge of node movement. The movement direction is determined by the various road directions of the node’s beginning place. VACaMobil compares the current number of cars in the simulation to the goal number of vehicles at each step of the mobility simulation. Depending on whether it is larger or lower than the target value, VACaMobil either waits for the number of vehicles to reduce toward the target value or, in the last case, the second scenario involves gradually adding more vehicles to the mobility simulation, raising the current value until the desired value is obtained. Output of the simulation contains several statistics for each car such as sent packets, received broadcasted packets, lost packets, speed, position, etc. Implementation of techniques follows the proposed frameworks due to the global nature of the problem for developed and underdeveloped countries. The results allow us to conclude that the implemented model is more suitable for simulating global network traffic simulation for estimating the traffic density on local ones. If the simulation is supposed to be used to evaluate such characteristics of a real network, then it is worth using models with an approximation not only of self-similar behavior, but also at least one-dimensional distribution.

## 8. Conclusions

This paper presented a comprehensive investigation for traffic density estimation techniques in the context of sustainable characteristics of intelligent transportation systems. The new approach is also introduced by constructing a Markov-based efficient model on OMNET++. The proposed and implemented model results are discussed in Table 5, and a critical number of vehicles equipped with V2X communication is required to significantly improve traffic efficiency. Moreover, the illustration is shown in the form of graphs as mentioned in Figure 9a–e, and Table 4 and Table 6 of the proposed model was successfully implemented with the V2X communication. The results from this research are utilized to gain a better understanding of how well information may be propagated over V2X in any conditions, as well as the properties of communication devices (in terms of communication range), which may be employed to accomplish specific system performance. This study’s model can simply be extended to help or predict the distance between successive roadside stations, vehicles, and obstacles. In this study, we also explain the “Markov traffic”-based mathematical model that is developed by using the graph theory and Markov modeling. The objective was to develop a method capable of estimation the steady state of traffic distribution over an urban traffic road network. This model is helpful in improving the urban traffic road architecture, which is one of its intended applications. As shown by our results, we are able to formulate an accurate estimation of the traffic density by tracking the vehicle velocity, acceleration, mean speed, and location of each and every vehicle. This model incorporates the factor that it produces a steady-state headways distribution observed in practice. Moreover, it is a microsimulation model that can mimic the transient-state statistics of road traffic. Implementation of this proposed model is in software to evaluate the approach. We have performed simulations by using both, i.e., the model and the proposed estimation method. Future research will focus on a comprehensive evaluation of the effects of each parameter on traffic efficiency. This study could concentrate on increasing the accuracy and effectiveness of the proposed model by optimizing the parameters and selecting a more effective imputed technique for corrupted data, among other things. 

## Figures and Tables

**Table 1:** Assessment of Simulation tools of TDE.

Type	Reference	Tools	Features	Language	Communication
Microscopic	[4]	AIMSUN	Precise signs, complicated traffic, signage, 3D animation, and telematics	C++	V2I & V2V
	[5]	PARAMICS	Route processes, Three-dimensional animation, junctions, and overloaded networks	C	V2I & V2V
	[6]	VISSIM	Three-dimensional animation, slope, transportation processes.	C	V2I & V2V
	[7]	MITSIM Lab	ATIS and ATMS.	C++	V2I & V2V
	[8]	CORSIM	Actuated signals, weaving sections, variable message signs, 2D animation, highways	C++	V2I & V2V
Macroscopic	[9]	SATURN	Individual junctions, for highway models	C	V2I & V2V
	[10]	KRONOS	Development of lines and congestion dissemination on and off the path.	C	V2V
	[11]	TRANSYT	Phase split, and cycle length of the traffic signals	C++	V2X
	[12]	KWaves	Throughput, bottlenecks, queues, ramp metering, incident management	C++	V2I
Mesoscopic	[13]	CORFLO	Surface streets, freeways, freeway corridor	C	V2I & V2V
	[14]	Micmac	Predicts the flow density, speed is used	C++	V2X
	[15]	Hystra	Based on traffic flow theory		V2I
	[16]	Integration	Ground roads, highways, road allocations, smart transport.	C++	V2I & V2X
	[17]	DynaMIT	Dynamic network state estimation, a number of ET scenarios	C++	V2I & V2X

**Table 2:** Traffic density estimation techniques.

Reference	Year	Publisher	Calibration of TDE Methods	Major Parameters for Calibration
[2]	2020	IEEE	Kernel density estimation and hotspot analysis	Vehicle sensors
[3]	2017	ELSEVIER	Comparison on simulation approach	Road location
[18]	2019	IEEE	Asymmetric cell transmission model	Loop detector
[19]	2019	IEEE	Kalman filtering technique	GPS & LiDAR
[20]	2019	IEEE	CNN model-based image recognition	Camera & speed measurement
[21]	2019	IEEE	Artificial neural network	Region of interest (ROI) & traffic video
[22]	2019	ELSEVIER	Mathematical model	Real traffic video
[23]	2019	MDPI	Mesoscopic traffic model	Inflow and outflow of Cell Transmission Model
[24]	2019	ELSEVIER	Hybrid observer (HO)-based exponentially weighted moving average-generalize likelihood ratio (EWMA-GLR)	exponentially weighted moving average (EWMA) scheme
[25]	2019	ELSEVIER	Single-shot detection (SSD) and mobile net-SSD	Number of images
[26]	2019	IEEE Journal	Single-state noncontinuum macroscopic model with Kalman filtering	EWMA scheme
[27]	2019	ELSEVIER	Interacting multiple model (IMM) filtering approach with a cell transmission model	GPS and in vehicle sensors
[28]	2019	IEEE Journal	Convex optimization technique	Traffic sensors
[29]	2018	IEEE journal	Real-time android application	Real-time live video
[30]	2015	IEEE journal	Onboard camera with linear parabolic lane mode	Onboard vehicular camera
[31]	2013	ELSEVIER	Macroscopic approach	K nearest neighbor (KNN)
[32]	2017	Turkish Journal	Kernel density estimation (KDE)	Speed center and the variance
[33]	2016	IEEE journal	Block based holistic approach	Vehicle in blocks, blocks of interest
[34]	2016	IEEE journal	Visible infrared imaging audiometer suite (VIIRS) sensor	Vessel detection
[35]	2016	ELSEVIER	Contiguous feature vector frames	Omnidirectional microphone

**Table 3:** Assessment of TDE methods.

Reference	Method	Scenario	Procedure	Algorithm	Approach
[24]	Sensor’s data	Highway	Live time	GLR	Static
[25]	Vehicle length for heterogeneity	Urban	VISSIM	Kalman Filter	Dynamic
[26]	Convex optimization	Highway	SDPNAL+	Lipschitz	Dynamic
[27]	Vehicle flow	Urban	Live time	ROI	Static
[28]	Vehicles data	Urban	Live time	Kernel density	Static
[29]	Vehicle counting	Urban	Live time	Block variance	Static
[30]	Images	Urban	Live time	eSNN	Dynamic
[31]	Images	Urban	Live time	CNN	Dynamic
[32]	Speed measurements	Highway	Aimsun	Kalman filter	Dynamic
[33]	Link count data	Urban	Live time	DBN	Dynamic
[34]	Measurements from a vehicle	Highway	Live time	Point of observations	Dynamic
[35]	Counting the vehicle number	Simulation	Live time	PON	Static
[36]	Vehicle trajectory data	Urban	VISSIM	Bayesian network	Static
[37]	Vehicle trajectories	Urban	Live time	Kalman–Bucy	Static
[26]	Loop detectors	Urban	Live video	Image processing	Dynamic
[38]	Images	Urban	Live time	KNN+ANN	Dynamic
[25]	Vehicles count	Highway	Live time	Single-shot Detection	Dynamic
[39]	Vehicles count	Highway	Live time	Smartphone GPS tracking	Dynamic
[40]	Infrared sensors	Urban	Live time	KNN	Dynamic
[41]	MATLAB	Highway	Live time	HMBLBP	Dynamic
[4]	Connected vehicle technology (CVT) statistics with (AI)	Urban	Live time	SPUI and TUDI	Dynamic

**Table 4:** Results of Markov based TDE.

Event Count	Time Duration	RSU Beacon ID	Packet ID	Car ID	Communication Type
1–82	0–84	6	446, 447	1	RSU to Node
85–474	84.000001 –88.36263944067	12–13,18–19 24–25,30–31 36–37,42–43	520–523 531–541 560–569 578–579	1,14,20 26,32,38	Node to Node Node to RSU RSU to Node
475–637	89–250	6,17	447,463	1	RSU to CU

**Table 5:** Simulation parameters.

Parameters	Values
Simulation time	250 s
Number of nodes	05
Beacon interval	1 s
Packet length	80 bits
Bit Rate	6 Mbps
Power level	68 dBm
Antenna distance with Node to RSU	1.895 m

**Table 6:** Traffic Contribution.

Vehicle Attributes	Count	Acceleration	Standard Deviation	Mean
	0	2414.847688	0.68132993	0.03416567
	0.1	2411.824329	0.66303203	0.036483
VACaMobil	0.2	2407.159224	0.67172648	0.03862666
	0.3	2402.288222	0.74833056	0.0398694
	0.4	2396.139437	0.6548475	0.03405378

## Data Availability

Not applicable.

## References

[1] Kwon J., Murphy K. (2000). Modeling Freeway Traffic with Coupled HMMs.

[2] Guerrero-Ibáñez J., Zeadally S., Contreras-Castillo J. (2018). Sensor technologies for intelligent transportation systems. Sensors.

[3] Mahmud S.S., Ferreira L., Hoque M.S., Tavassoli A. (2019). Micro-simulation modelling for traffic safety: A review and potential application to heterogeneous traffic environment. IATSS Res..

[4] Rahimi A.M., Rahimi F. (2020). Traffic Evaluation of SPUI and TUDI Interchanges Based on Traffic Conditions in Tehran with Aimsun and Synchro Software. Iran. J. Sci. Technol. Trans. Civ. Eng..

[5] Nam D., Lavanya R., Jayakrishnan R., Yang I., Jeon W.H. (2020). A deep learning approach for estimating traffic density using data obtained from connected and autonomous probes. Sensors.

[6] Sun D.J., Zhang L., Chen F. (2013). Comparative study on simulation performances of CORSIM and VISSIM for urban street network. Simul. Model. Pract. Theory.

[7] Kumar S.V., Vanajakshi L., Subramanian S.C. (2011). Location-based data for estimated traffic on Urban arterial in heterogeneous traffic conditions. Transp. Res. Rec..

[8] Park S., Sohn S., Jae M. (2021). Cohort-based evacuation time estimation using TSIS-CORSIM. Nucl. Eng. Technol..

[9] Oskarbski J., Kaszubowski D. (2018). Applying a mesoscopic Transport Model to analyse the effects of urban freight regulatory measures on transport emissions—An assessment. Sustainability.

[10] Ratrout N.T., Rahman S.M. (2009). A comparative analysis of currently used microscopic and macroscopic traffic simulation software. Arab. J. Sci. Eng..

[11] Chiou S.W. (2010). An efficient algorithm for computing traffic equilibria using TRANSYT model. Appl. Math. Model..

[12] Daganzo C.F. (2005). A variational formulation of kinematic waves: Basic theory and complex boundary conditions. Transp. Res. Part B Methodol..

[13] Lieu H., Santiago A., Kanaan A. Corflo. An integrated traffic simulation system for corridors. Proceedings of the Traffic Management, Engineering Foundation ConferenceEngineering Foundation.

[14] Kadam S., Bandyopadhyay P.K. (2020). Modelling passenger interaction process (PIP) framework using ISM and MICMAC approach. J. Rail Transp. Plan. Manag..

[15] Bourrel E., Lesort J.B. (2003). Mixing microscopic and macroscopic representations of traffic flow: Hybrid model based on Lighthill–Whitham–Richards theory. Transp. Res. Rec..

[16] Dorokhin S., Artemov A., Likhachev D., Novikov A., Starkov E. (2020). Traffic simulation: An analytical review. IOP Conference Series: Materials Science and Engineering.

[17] Reyes-Rubiano L., Serrano-Hernandez A., Montoya-Torres J.R., Faulin J. (2021). The Sustainability Dimensions in Intelligent Urban Transportation: A Paradigm for Smart Cities. Sustainability.

[18] Ali S.S.M., George B., Vanajakshi L., Venkatraman J. (2011). A multiple inductive loop vehicle detection system for heterogeneous and lane-less traffic. IEEE Trans. Instrum. Meas..

[19] Sochor J., Juránek R., Špaňhel J., Maršík L., Širokỳ A., Herout A., Zemčík P. (2018). Comprehensive data set for automatic single camera visual speed measurement. IEEE Trans. Intell. Transp. Syst..

[20] Sochor J., Juránek R., Herout A. (2017). Traffic surveillance camera calibration by 3d model bounding box alignment for accurate vehicle speed measurement. Comput. Vis. Image Underst..

[21] Tan E., Chen J. Vehicular traffic density estimation via statistical methods with automated state learning. Proceedings of the 2007 IEEE Conference on Advanced Video and Signal Based Surveillance.

[22] Ozkurt C., Camci F. (2009). Automatic traffic density estimation and vehicle classification for traffic surveillance systems using neural networks. Math. Comput. Appl..

[23] Panda M., Ngoduy D., Vu H.L. (2019). Multiple model stochastic filtering for traffic density estimation on urban arterials. Transp. Res. Part B Methodol..

[24] Zeroual A., Harrou F., Sun Y. (2019). Road traffic density estimation and congestion detection with a hybrid observer-based strategy. Sustain. Cities Soc..

[25] Biswas D., Su H., Wang C., Stevanovic A., Wang W. (2019). An automatic traffic density estimation using Single Shot Detection (SSD) and MobileNet-SSD. Phys. Chem. Earth Parts A/B/C.

[26] George R., Vanajakshi L.D., Subramanian S.C. (2019). Area Occupancy-Based Adaptive Density Estimation for Mixed Road Traffic. IEEE Access.

[27] Jo K., Chu K., Sunwoo M. (2011). Interacting multiple model filter-based sensor fusion of GPS with in-vehicle sensors for real-time vehicle positioning. IEEE Trans. Intell. Transp. Syst..

[28] Nugroho S.A., Taha A.F., Claudel C.G. (2019). A control-theoretic approach for scalable and robust traffic density estimation using convex optimization. IEEE Trans. Intell. Transp. Syst..

[29] Kerouh F., Ziou D. (2018). Real-time Android application for traffic density estimation. IEEE Access.

[30] de Paula M.B., Jung C.R. (2015). Automatic detection and classification of road lane markings using onboard vehicular cameras. IEEE Trans. Intell. Transp. Syst..

[31] Asmaa O., Mokhtar K., Abdelaziz O. (2013). Road traffic density estimation using microscopic and macroscopic parameters. Image Vis. Comput..

[32] Yilan M., Özdemir M.K. (2017). Traffic density estimation via KDE and nonlinear LS. Turk. J. Electr. Eng. Comput. Sci..

[33] Garg K., Lam S.K., Srikanthan T., Agarwal V. Real-time road traffic density estimation using block variance. Proceedings of the 2016 IEEE Winter Conference on Applications of Computer Vision (WACV).

[34] Yamaguchi T., Asanuma I., Park J.G., Mackin K.J., Mittleman J. Estimation of vessel traffic density from Suomi NPP VIIRS day/night band. Proceedings of the Oceans 2016 MTS/IEEE Monterey.

[35] Borkar P., Sarode M., Malik L. (2016). Employing speeded scaled conjugate gradient algorithm for multiple contiguous feature vector frames: An approach for traffic density state estimation. Procedia Comput. Sci..

[36] Zhong Z., Lee E.E., Nejad M., Lee J. (2020). Influence of CAV clustering strategies on mixed traffic flow characteristics: An analysis of vehicle trajectory data. Transp. Res. Part C Emerg. Technol..

[37] Xie G., Gao H., Qian L., Huang B., Li K., Wang J. (2017). Vehicle trajectory prediction by integrating physics-and maneuver-based approaches using interactive multiple models. IEEE Trans. Ind. Electron..

[38] Raj J., Bahuleyan H., Vanajakshi L.D. (2016). Application of data mining techniques for traffic density estimation and prediction. Transp. Res. Procedia.

[39] Martínez-Díaz M., Soriguera F. (2021). Short-term prediction of freeway travel times by fusing input-output vehicle counts and GPS tracking data. Transp. Lett..

[40] Warriach E.U., Claudel C. A machine learning approach for vehicle classification using passive infrared and ultrasonic sensors. Proceedings of the 2013 ACM/IEEE International Conference on Information Processing in Sensor Networks (IPSN).

[41] Hu H., Gao Z., Sheng Y., Zhang C., Zheng R. (2019). Traffic density recognition based on image global texture feature. Int. J. Intell. Transp. Syst. Res..

[42] Lozano Domínguez J.M., Al-Tam F., Mateo Sanguino T.d.J., Correia N. (2020). Analysis of Machine Learning Techniques Applied to Sensory Detection of Vehicles in Intelligent Crosswalks. Sensors.

[43] Mukherjee A., Jain D.K., Goswami P., Xin Q., Yang L., Rodrigues J.J. (2020). Back propagation neural network based cluster head identification in MIMO sensor networks for intelligent transportation systems. IEEE Access.

[44] Goswami P., Mukherjee A., Hazra R., Yang L., Ghosh U., Qi Y., Wang H. (2021). AI based energy efficient routing protocol for intelligent transportation system. IEEE Trans. Intell. Transp. Syst..

[45] Gupta M., Kumar R., Shekhar S., Sharma B., Patel R.B., Jain S., Dhaou I.B., Iwendi C. (2022). Game Theory-Based Authentication Framework to Secure Internet of Vehicles with Blockchain. Sensors.

[46] Gupta M., Patel R.B., Jain S., Garg H., Sharma B. (2022). Lightweight branched blockchain security framework for Internet of Vehicles. Trans. Emerg. Telecommun. Technol..

[47] Garg S., Singh R., Obaidat M.S., Bhalla V.K., Sharma B. (2020). Statistical vertical reduction-based data abridging technique for big network traffic dataset. Int. J. Commun. Syst..

[48] Zhang Y., Fricker J.D. (2020). Multi-state semi-Markov modeling of recurrent events: Estimating driver waiting time at semi-controlled crosswalks. Anal. Methods Accid. Res..

[49] Singh K., Li B. (2011). Estimation of traffic densities for multilane roadways using a markov model approach. IEEE Trans. Ind. Electron..

[50] Wang X., Peng L., Chi T., Li M., Yao X., Shao J. (2015). A hidden Markov model for urban-scale traffic estimation using floating car data. PLoS ONE.

[51] Priambodo B., Ahmad A., Kadir R.A. (2021). Predicting Traffic Flow Propagation Based on Congestion at Neighbouring Roads Using Hidden Markov Model. IEEE Access.

[52] Zaki J.F., Ali-Eldin A., Hussein S.E., Saraya S.F., Areed F.F. (2020). Traffic congestion prediction based on Hidden Markov Models and contrast measure. Ain Shams Eng. J..

[53] Báguena M., Tornell S.M., Torres Á., Calafate C.T., Cano J.C., Manzoni P. Vacamobil: Vanet car mobility manager for omnet++. Proceedings of the 2013 IEEE International Conference on Communications Workshops (ICC).

